# Genomic and Phenotypic Characterization of Mastitis-Causing Staphylococci and Probiotic Lactic Acid Bacteria Isolated from Raw Sheep’s Milk

**DOI:** 10.3390/ijms241813883

**Published:** 2023-09-09

**Authors:** Ilias Apostolakos, Theodora Skarlatoudi, Kornilia Vatavali, Agathi Giannouli, Loulouda Bosnea, Marios Mataragas

**Affiliations:** Department of Dairy Research, Institution of Technology of Agricultural Products, Hellenic Agricultural Organization “DIMITRA”, 3rd Ethnikis Antistaseos St., 45221 Ioannina, Greece; iaposto@hotmail.com (I.A.); theodoraskarlatoudi@hotmail.com (T.S.); kvatavali26@gmail.com (K.V.); agigiannouli@yahoo.gr (A.G.); louloudabosnea@gmail.com (L.B.)

**Keywords:** sheep’s milk, dairy products, lactic acid bacteria, mastitis, probiotics, staphylococci, whole-genome sequencing

## Abstract

Dairy products play a crucial role in human nutrition as they provide essential nutrients. However, the presence of diverse microorganisms in these products can pose challenges to food safety and quality. Here, we provide a comprehensive molecular characterization of a diverse collection of lactic acid bacteria (LAB) and staphylococci isolated from raw sheep’s milk. Whole-genome sequencing, phenotypic characterization, and bioinformatics were employed to gain insight into the genetic composition and functional attributes of these bacteria. Bioinformatics analysis revealed the presence of various genetic elements. Important toxin-related genes in staphylococci that contribute to their pathogenic potential were identified and confirmed using phenotypic assays, while adherence-related genes, which are essential for attachment to host tissues, surfaces in the dairy environment, and the creation of biofilms, were also present. Interestingly, the *Staphylococcus aureus* isolates belonged to sequence type 5, which largely consists of methicillin-susceptible isolates that have been involved in severe nosocomial infections. Although genes encoding methicillin resistance were not identified, multiple resistance genes (RGs) conferring resistance to aminoglycosides, macrolides, and fluroquinolones were found. In contrast, LAB had few inherently present RGs and no virulence genes, suggesting their likely safe status as food additives in dairy products. LAB were also richer in bacteriocins and carbohydrate-active enzymes, indicating their potential to suppress pathogens and effectively utilize carbohydrate substrates, respectively. Additionally, mobile genetic elements, present in both LAB and staphylococci, may facilitate the acquisition and dissemination of genetic traits, including RGs, virulence genes, and metabolic factors, with implications for food quality and public health. The molecular and phenotypic characterization presented herein contributes to the effort to mitigate risks and infections (e.g., mastitis) and enhance the safety and quality of milk and products thereof.

## 1. Introduction

Dairy food products play a key role in the human diet as they provide a necessary source of high-value nutrients, including proteins, vitamins, and minerals [1]. Their safety and quality are largely affected by the various microorganisms present. Two of the major groups of bacteria found in dairy products are of great interest due to their diverse metabolic activities and potential impact on dairy product characteristics and safety.

The first group are lactic acid bacteria (LAB), which are Gram-positive, non-sporulating bacteria that are widely distributed in various ecological niches [2]. They contribute to milk fermentation, ensuring desirable flavor and texture in fermented milk-based products, and thus play a key role in the dairy industry [3]. Moreover, they produce lactic acid as a major catabolic product from lactose, which reduces the pH and thereby inhibits the growth of pathogenic bacteria [3]. Furthermore, studies show that LAB produce antimicrobial substances than can further contribute to the preservation of dairy foods [4].

The second group are staphylococci (coagulase-negative and -positive strains), which are Gram-positive, non-motile cocci found in abundance on the skin and mucosal surfaces of humans and animals. Staphylococci impact both animal health and the quality of dairy foods, and they are often isolated from dairy environments. Although some species are used as starter cultures for the production of fermented products (e.g., *Staphylococcus xylosus*), others can cause infections and foodborne diseases and therefore pose risks to consumer health and livestock [5]. *Staphylococcus aureus*, for example, causes intramammary, skin, and respiratory infections as well as food poisoning [6]. Hence, it is of critical importance to understand the genetic traits of staphylococci in dairy environments to ensure animal health and food safety. 

The development of molecular techniques such as whole-genome sequencing and bioinformatics has advanced our understanding of microbial populations, providing valuable insight into the genetic structure and functional potential of microorganisms present in complex environments. Multiple studies have investigated various aspects of LAB and staphylococci in dairy foods (e.g., [4,5,7]), providing insight into the functional diversity within each species.

We previously investigated the functional traits and safety status of various LAB [8,9] and enterococci [10] isolated from artisanal cheeses. The aim of the present study was to expand the existing body of knowledge by performing a comprehensive molecular characterization of the LAB and coagulase-negative and -positive staphylococcal strains isolated from raw sheep’s milk. Genetic characterization of the strains was accompanied with phenotypic assays to confirm their genetic potential. Our main goal was to elucidate the presence and diversity of antimicrobial resistance and virulence genes, the phylogenetic relationships within the LAB and staphylococcal populations, as well as the functional diversity and potential metabolic capabilities of these groups.

## 2. Results and Discussion

### 2.1. Assembly Statistics

Table 1 presents information regarding the species identification, isolation source, and assembly statistics for each genome. Statistical analysis was conducted to assess the fundamental characteristics of the LAB genomes. The average size of the genomes in this collection was 2.31 Mbp, with *Staphylococcus aureus* strains having the largest average genome of 2.78 Mbp. The genomes of *S. aureus* strains were significantly larger (*p* ≤ 0.05) than those of *Lactococcus lactis* (+0.48 Mbp, on average), *Staphylococcus simulans* (+0.54 Mbp), *Lactococcus petauri* (+0.83 Mbp), *Lactobacillus delbrueckii* subsp. *lactis* (+ 0.88 Mbp), and *Limosilactobacillus fermentum* (+0.94 Mbp). In terms of GC content, the average GC content was 39.94%, with *Lmb. fermentum* (52.67%) and *Staphylococcus (Mammaliicoccus) lentus* (31.79%) having the significantly largest and smallest GC contents (*p* ≤ 0.05), respectively. Moreover, the average number of coding sequences (CDSs) was 2194. 

### 2.2. Lactic Acid Bacteria

#### 2.2.1. Phylogenetic Comparison and Functional Analysis

**Figure 1:**
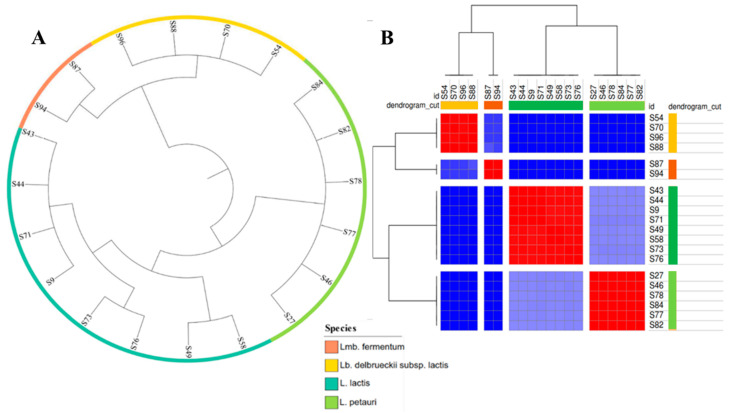
Phylogenetic tree (**A**) and heatmap of ANI values (**B**) of the 20 LAB isolates. The colored ring designates the species of the isolates according to the legend, while the colored squares designate the microorganisms’ relatedness based on their ANI values (red color > the threshold value of 95–96%, dark and light blue < the threshold value of 95–96%).

The phylogenetic tree of LAB is shown in Figure 1A. Overall, the combined gene pool of the LAB genomes contained 8103 clusters of orthologous genes (COGs), while the fundamental set of genes common to ≥90% of all LAB isolates (core genome) consisted of 21 COGs. By constructing a phylogenetic tree via alignment of the core genes, we were able to discern the genomic similarities among the investigated LAB isolates (Figure 1A). The isolates formed distinct clusters based on their assigned species, with no instances of cluster overlap observed. The calculated average nucleotide identity (ANI) values (Figure 1B) supported the isolate clustering using a threshold value of 95–96% for species delineation.

**Figure 2:**
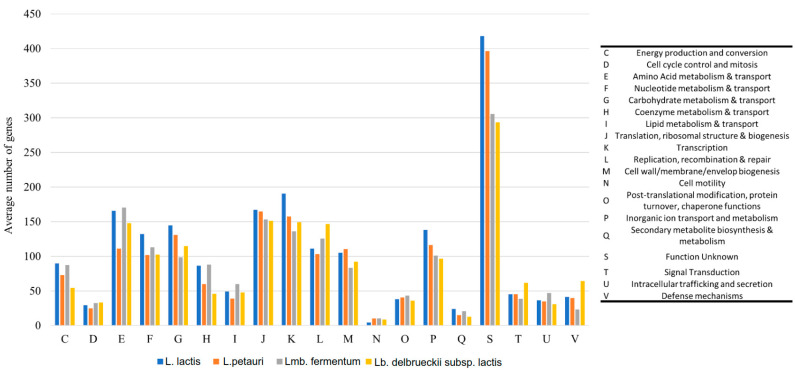
Distribution of COG functional categories among LAB.

A subsystem comprises a group of protein-coding sequences (CDSs) that collectively perform a distinct biological function or form a structural complex [11]. Analysis of the subsystems in the LAB genomes identified the presence of 19 COG categories that exhibited enrichment (Figure 2). Among these categories, the COG designated as unknown function (S) displayed the highest degree of enrichment, with an average of 353 CDSs observed across the LAB genomes (19.6% of all COGs). Notably, *L. lactis* demonstrated significantly greater enrichment in this category, as well as in 5 additional categories out of the 19 examined, compared to the other LAB genomes (Figure 2). These categories were inorganic ion transport and metabolism (P, 6.3% of COGs), transcription (K, 8.8%), ribosomal structure and biogenesis (J, 8.8%), and carbohydrate metabolism and transport (G, 6.8%). These findings suggested that *L. lactis* possessed a highly functional genome while also harboring a substantial portion of CDSs with yet undetermined functions, which warrants further investigation and characterization [12].

#### 2.2.2. Virulence and Antimicrobial Resistance Determinants

Antimicrobial resistance genes (RGs) were generally infrequent in LAB. The chromosomally located ABC efflux pump encoded by the *lmrD* gene, which provides resistance to lincosamides in *Streptomyces lincolnensis* and *L. lactis* and its deletion renders the bacteria susceptible to toxic compounds [3], was found in all (*n* = 8) strains of *L. lactis*. Moreover, the *mdtA* gene was found in all strains of *L. petauri* (*n* = 6). This gene encodes a membrane fusion protein of the *mdtABC* resistance–nodulation–cell division (RND) multidrug efflux complex, which is a prevalent resistance mechanism in *Escherichia* and *Shigella* spp. [13]. Given that the other two components of the system (*mdtB* and *mdtC*) were absent, it is likely that the efflux pump was inactive in the *L. petauri* strains of our collection. Lastly, we did not identify any virulence genes (VGs) in the LAB.

#### 2.2.3. Bacteriocins and Carbohydrate-Active Enzymes (CAZymes)

In the in silico typing of LAB, we identified five genes encoding for bacteriocins in the genomes of *L. delbrueckii* subsp. *lactis* and *L. lactis* (*n* = 22). More specifically, in eight strains we found bovicin 255, a bacteriocin initially isolated from *Streptococcus bovis* inhabiting the rumen of herbivores that exhibits antimicrobial activity against a range of pathogens, such as *Enterococcus faecium* and *Clostridium perfringens* [14]. Moreover, enterolysin A was identified in eight strains. This bacteriocin, typically produced by *Enterococcus faecalis*, disrupts the cell membrane integrity of target bacteria (other enterococci), leading to cell lysis. Enterolysin A has potential applications in controlling enterococcal infections and combating antibiotic-resistant strains [15]. Finally, we found carnolysin and lactococcin B in two strains each. Carnolysin is a bacteriocin produced by *Carnobacterium maltaromaticum*, an LAB, which acts by disrupting the cell membrane integrity of target bacteria, leading to cell death. It has potent antimicrobial activity against *Listeria monocytogenes*, making it a potential natural alternative for controlling this foodborne pathogen [16]. Lactococcin B exhibits broad-spectrum antimicrobial activity against various Gram-positive bacteria, including *Listeria monocytogenes* [17]. These findings highlighted the potential of the analyzed LAB strains to produce antimicrobial substances that can contribute to their ecological fitness and potential applications in food preservation and probiotic formulations. Moreover, bacteriocin production by LAB may exert inhibitory effects on staphylococci, which could influence microbial competition and enhance the safety and quality of dairy products. Further experimental investigations on the identified bacteriocins are warranted to explore these potential interactions and their practical applications in dairy food production [18].

We found 23 distinct CAZymes clustered in five groups. Glycosyltransferases (GTs) were the most abundant class, making up 59% of all identified CAZymes. GTs are enzymes responsible for the transfer of sugar moieties; hence, they play an essential role in the biosynthesis of carbohydrates. Moreover, GTs participate in various biological processes, including the synthesis of cell wall polysaccharides, exopolysaccharides, as well as other complex carbohydrates [19]. Glycoside hydrolases (GHs) were also prevalent (29% of all CAZymes). These enzymes are involved in the breakdown of glycosidic bonds and catabolism of carbohydrates, such as starch, cellulose, and chitin. The abundance of GHs suggested that LAB possessed the enzymatic machinery to hydrolyze and utilize a broad spectrum of complex carbohydrates [20]. Lastly, carbohydrate-binding modules (CBMs) were found less frequently (8% of CAZymes). However, their presence indicated the potential for LAB to interact with and adhere to specific carbohydrate substrates, potentially facilitating their utilization and metabolism [21].

#### 2.2.4. Mobile Genetic Elements and Prophages

Although no plasmids were identified in the LAB genomes, we found eight insertion sequences (ISs). ISs are small, self-transferable mobile genetic elements that can move across bacterial genomes, playing a significant role in genome plasticity and adaptation to the environment [22]. *ISLll1*, the predominant IS identified in the LAB genomes, was found in two strains of *L. lactis* and four strains of *L. petauri*. According to the ISfinder database, this IS was first detected in 1992 by Durmaz et al. [23]; however, we found only one recent publication reporting its presence in a *L. petauri* strain from Montenegrin brine cheeses [24]. *ISLll1* belongs to the IS982 family, members of which are prevalent in LAB, and it is implicated in the acquisition and dissemination of RGs [25]. Notably, in the same IS family, we identified the representative element *IS982* (*n* = 3). Moreover, *ISLL6* (IS3 family), the second most prevalent IS (*n* = 4), is the most abundant IS element found in *L. garvieae* and has been associated with the rearrangement of LAB genomes through recombination events [26].

Seven prophages with intact genomes were identified in LAB, with *Lactococcus* phage 63301 (accession: NC_031017.1) being the most prevalent (*n* = 5 LAB genomes), followed by *Lactococcus* phage bIL310 (accession: AF323671.1) (*n* = 4). Overall, 16 out of 20 LAB strains had at least one intact prophage integrated in their genome. Specific information regarding these prophages is scarce; however, their presence in LAB from dairy products is significant for several reasons. From a safety and quality standpoint, prophages are contaminants, since they can be induced during fermentation under certain conditions, leading to cell lysis, which negatively impacts the final product [27]. On the other hand, studies have shown that prophages significantly enhance the genetic diversity and phenotypic plasticity of LAB and may enable their adaptation to various environmental conditions [28].

#### 2.2.5. Probiotic Status of the Isolated LAB Strains

**Figure 3:**
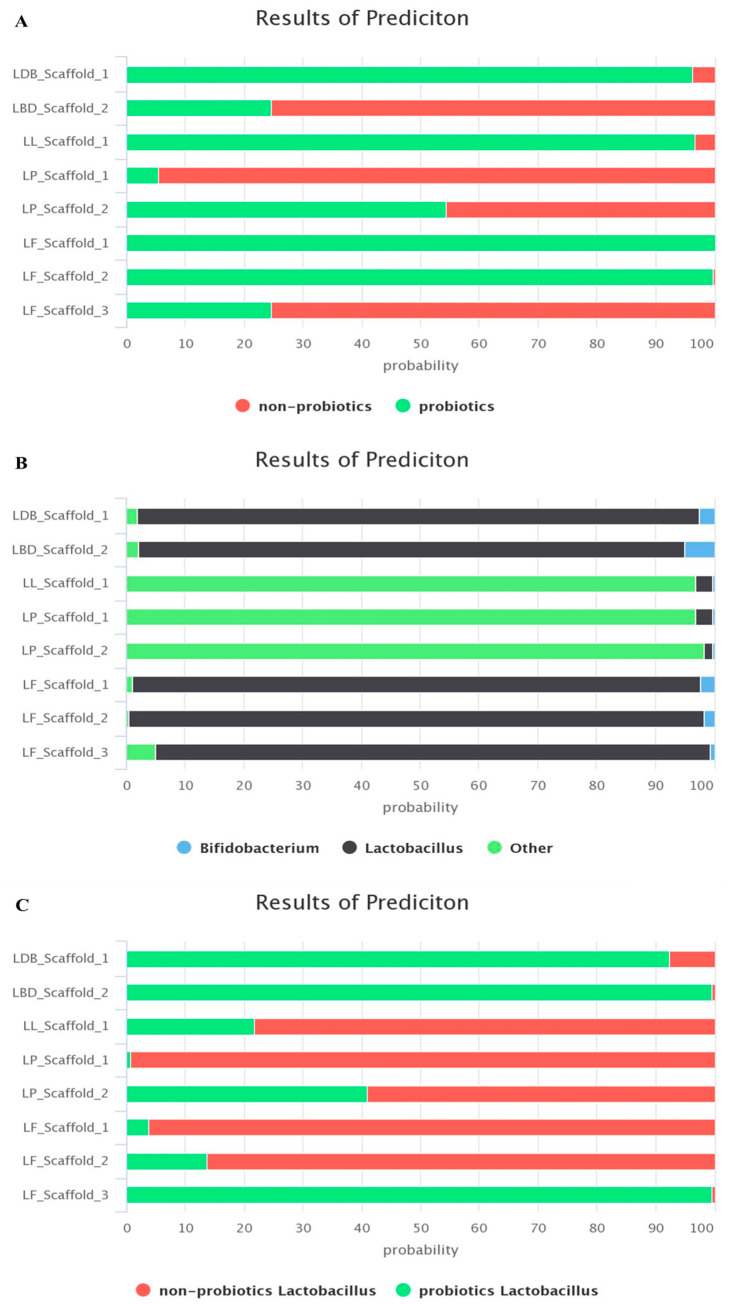
Stacked bar chart regarding LAB probiotic status as predicted by the iProbiotics webtool using the three available predictive SVM models: Probiotic Predictor (model 1) (**A**), Probiotic *Lactobacillus*, *Bifidobacterium* and other Predictor (model 2) (**B**), and Probiotic *Lactobacillus* Predictor (model 3) (**C**). LDB, *Lactobacillus delbrueckii* subsp. *bulgaricus*; LL, *Lactococcus lactis*; LP, *Lactococcus petauri*; LF, *Limosilactobacillus fermentum*.

LAB probiotic status was assessed using the probiotic predictor tool iProbiotics, which comprises three different models based on a support vector machine (SVM) algorithm and different datasets, namely Probiotic Predictor (model 1), Probiotic *Lactobacillus*, *Bifidobacterium,* and others Predictor (model 2), and Probiotic *Lactobacillus* Predictor (model 3). The LAB genomes were run with all available models and the results are presented in Figure 3.

The first generic model (Figure 3A) predicted that all LAB species had probiotic properties (probability > 95%) except *L. petauri*, which was a non-probiotic species (low probability). The other two models (Figure 3B,C) were more specific and showed that eventually only *Lb. delbrueckii* subsp. *bulgaricus* and *L. lactis* could be classified as probiotic cultures and considered as potential candidates for fighting mastitis-causing pathogens.

**Figure 4:**
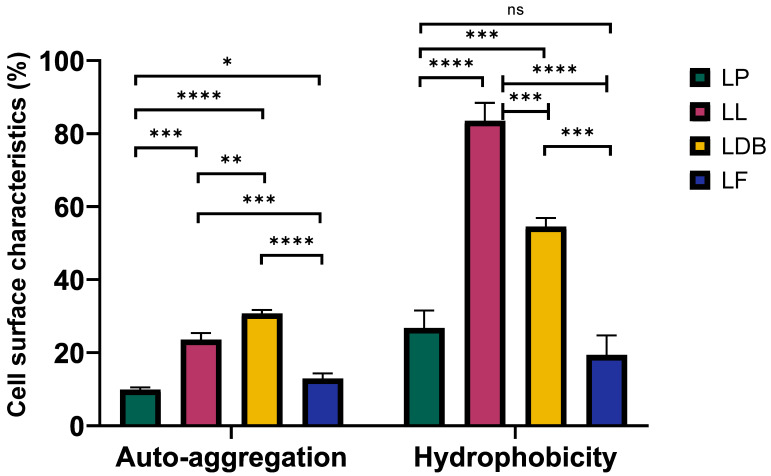
Percentage and statistical comparison of LAB species for cell surface hydrophobicity after 0.5 h and auto-aggregation after 5 h. ns, not significant, * *q* < 0.05, ** *q* < 0.01, *** *q* < 0.001, and **** *q* < 0.0001. LP, *Lactococcus petauri*; LL, *Lactococcus lactis*; LDB, *Lactobacillus delbrueckii* subsp. *bulgaricus*; LF, *Limosilactobacillus fermentum*.

One important parameter for preventing mastitis-causing pathogens is the adhesion capability of bacteria in order to successfully colonize the udder. Cell surface hydrophobicity (CSH) and auto-aggregation (AA) are the two cell surface properties that demonstrate the microbial adhesion ability [29]. Auto-aggregation increased with time for all strains (exception *Lmb. fermentum*, which showed a peak at 4 h, AA = 16.64%). *Lb. delbrueckii* subsp. *bulgaricus* presented the highest AA value (30.72 ± 0.94%) after 5 h, followed by *L. lactis* (23.51 ± 1.23%), while *L. petauri* and *Lmb. fermentum* showed the lowest AA values (10.98 ± 0.56% and 12.92 ± 1.75%, respectively) (Figure 4). A similar trend was observed for CSH. *L. lactis* had the optimal CSH value (83.49 ± 4.94%), followed by *Lb. delbrueckii* subsp. *bulgaricus* (54.53 ± 2.32%). Again, the CSH values of *L. petauri* and *Lmb. fermentum* were significantly lower (26.72 ± 4.83% and 19.39 ± 5.30%, respectively) than those of the other two strains. These results supported the predictions about the probiotic status of the LAB strains. Previously, it was found that the *Lb. delbrueckii* subsp. *bulgaricus* and *L. lactis* genomes contained genes encoding bacteriocins (antagonistic activity against pathogens) and genes related to probiotic traits (Table 2). In addition, these two microorganisms showed the best CSH and AA values, which aligned with the adhesion-related genes found in their genomes (Table 2). Therefore, the two species are suitable for potential application as protective probiotic cultures.

### 2.3. Staphylococci

#### 2.3.1. Phylogenetic Analysis and Comparative Genomics

**Figure 5:**
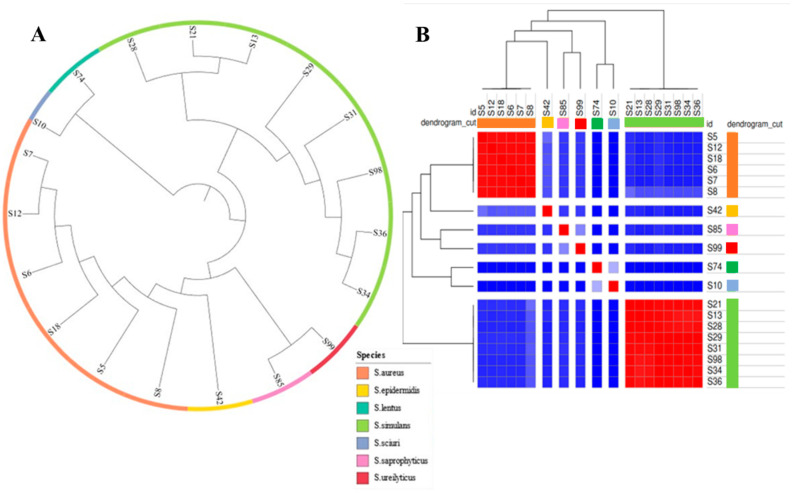
Phylogenetic tree (**A**) and heatmap of the average nucleotide identity (ANI) values (**B**) of the 19 staphylococcal isolates. The colored ring designates the species of the isolates according to the legend, while the colored squares designate the microorganisms’ relatedness based on their ANI values (red color > the threshold value of 95–96%, dark and light blue < the threshold value of 95–96%).

The phylogenetic comparison of staphylococci is shown in Figure 5A. Overall, the combined gene pool of the staphylococcal genomes contained 10,717 COGs, while the core genome (at a 90% threshold) had 323 COGs. By constructing a phylogenetic tree via core genome alignment, we identified the genomic similarities among staphylococci (Figure 5A). As with the phylogenetic tree of the LAB strains, the *Staphylococcus* spp. isolates formed distinct clusters based on their assigned species, while no cluster overlap was observed. Similarly, the estimated ANI values (Figure 5B) confirmed the clustering of staphylococci into different species (species delineation threshold value equal to 95–96%).

The *mecA*/*mecC* genes were not detected in all *S. aureus* and coagulase-negative staphylococcal (CNS) strains; thus, they were designated as methicillin-susceptible (MSSA) microorganisms. All *S. aureus* strains were predicted to be human pathogens (probability 98.4%), as they carried the vSa-gamma (*vSaγ*) pathogenicity island (*SaPI*) and other mobile genetic elements (MGEs), with genes related to adherence, antiphagocytosis, exoenzyme, hostimm, immune evasion, iron uptake, plasminogen activation, the secretion system, and toxin production. The CNS strains were also predicted to be human pathogens (probability ranged from 63.8% to 98.5%). *S. simulans* displayed a much lower probability of being a human pathogen (82% on average) than other staphylococci, such as *S. aureus* (98.4%), *S. saprophyticus* (97.5%), *S. epidermidis* (95.4%), and *S. ureilyticus* (98.5%). Indeed, although *S. simulans* can cause persistent intramammary infections as well as increased somatic cell counts to values comparable to those of *S. aureus*, the infection and incurred symptoms are less severe (e.g., no clinical mastitis) compared to other mastitis-causing staphylococci (e.g., *S. aureus*) [30,31]. Additionally, the *S. aureus* strains belonged to sequence type (ST) 5 (clonal complex 5) and *spa* type t306. Notably, this ST has been implicated in severe nosocomial infections and is usually recovered from human blood isolates; however, it seems to largely consist of methicillin-susceptible isolates [32]. It is worth mentioning that the MLST analysis detected a point mutation in the *glpF* gene at the base pair position 438 (A instead of T), which may indicate a novel ST close to ST5, but further experimental confirmation is required, e.g., sequencing of this specific gene fragment using the Sanger technique.

**Figure 6:**
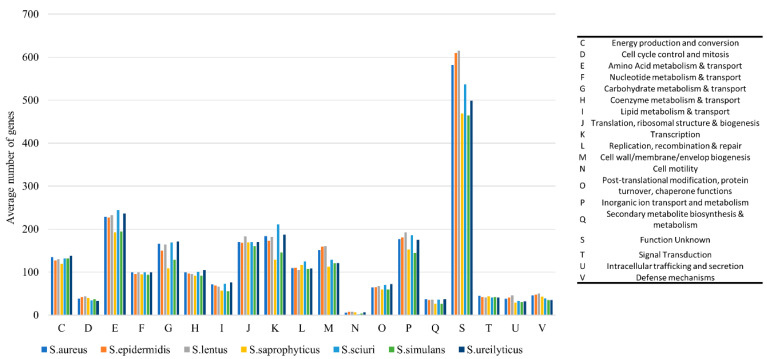
Distribution of COG functional categories among staphylococci.

Analysis of the subsystems present in the *Staphylococcus* spp. genomes revealed the presence of 19 COG categories that exhibited enrichment (Figure 6). The CDSs with unknown function (S) were the most enriched, with an average of 540 CDSs observed across the staphylococcal genomes (24% of all COGs). Other enriched COG categories were amino acid metabolism and transport (E) with 222 CDSs (9.5%); transcription (K) and inorganic ion transport/metabolism (P), each with 173 CDSs (7.4%); as well as ribosomal structure and biogenesis with 170 CDSs (7.3%). The rest of the COG categories together made less than half (45.2%) of all COGs (Figure 6). Notably, our statistical analysis showed a significant difference in the enrichment of only one COG, secondary metabolite biosynthesis (Q), for which *S. aureus* was significantly more enriched than *S. simulans* (11 more CDSs, on average).

#### 2.3.2. Virulence and Antimicrobial Resistance Determinants

**Figure 7:**
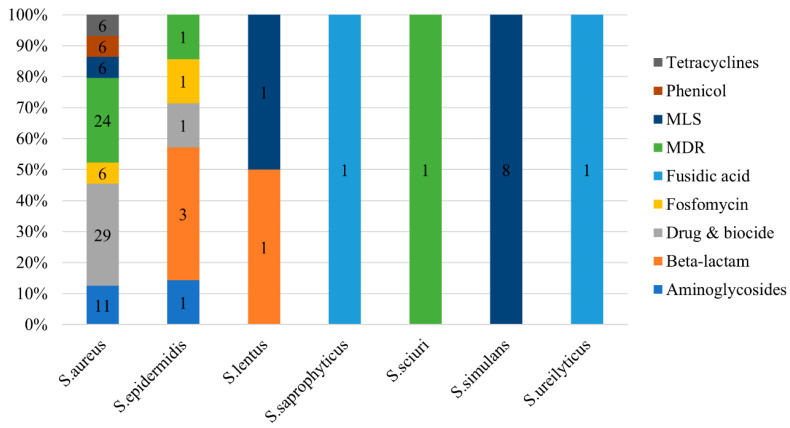
Distribution of antimicrobial resistance genes (ARGs) in seven species of staphylococci. The type of resistance is colored according to the legend. Data labels in the center of bars indicate the number of genes found in the corresponding species and type of resistance.

Our analysis revealed the presence of 108 RGs in staphylococci (Figure 7). Most RGs (81.5%) were found in *S. aureus*, followed by *S. simulans* (7.4%) and *Staphylococcus epidermidis* (6.5%). Resistance to drugs and biocides was the most prevalent type of resistance (27.8% of all RGs), primarily represented by the *norA* and *norB* genes (5.6%, each). *norA* encodes a well-studied efflux system in *S. aureus* and serves as a model for understanding efflux-mediated resistance. Additionally, it contributes to resistance against fluoroquinolones, antiseptics, and disinfectants [33]. Similarly, *norB* encodes a multidrug efflux pump providing resistance to fluroquinolones that is, however, independent of *norA* [34]. Furthermore, the *arlR*-*arlS* locus, which regulates the (over)expression and multidrug resistance (MDR) phenotype of *norA*-carrying *S. aureus* strains [35], was identified in six strains (11.2%) of this species. Another gene found in the MDR class of resistance was the regulator *mgrA*, which controls *norA*, *norB*, as well as *tet38* [36].

In the macrolides, lincosamides, and streptogramines (MLS) category (13.9% of all RGs), we found ribosomal large-subunit methyltransferase H (*rlmH*, also known as *orfX* [37]) in all strains of *S. aureus* (*n* = 6) and *S. simulans* (*n* = 8). Except from its action in preventing the activity of the MLS group of antibiotics, the *rlmH* gene, found in all staphylococci, remains unaffected even after insertion of the staphylococcal chromosome cassette *mec* (SCCmec). This cassette carries the *mecA* gene, which is responsible for encoding resistance to β-lactam antibiotics, and it is inserted at the C terminus of the *rlmH* gene [37]. Notably, we did not detect any *mec* genes (*mecA*, *mecB*, *mecC*) in our collection of staphylococci.

Genes conferring resistance to aminoglycosides were also found (11.1% of RGs). This category includes N-acetyltransferase *aac(3′)* and O-phosphotransferase *aph(3′)*. Other resistance classes were less common (Figure 7). We identified *tet38*, which encodes a tetracycline efflux pump, in the strains of *S. aureus* (*n* = 6), whereas *blaZ*, which encodes β-lactamase (penicillinase), was found in one strain each of *S. lentus* and *S. epidermidis*. 

All identified RGs are listed in Supplementary Table S1.

**Figure 8:**
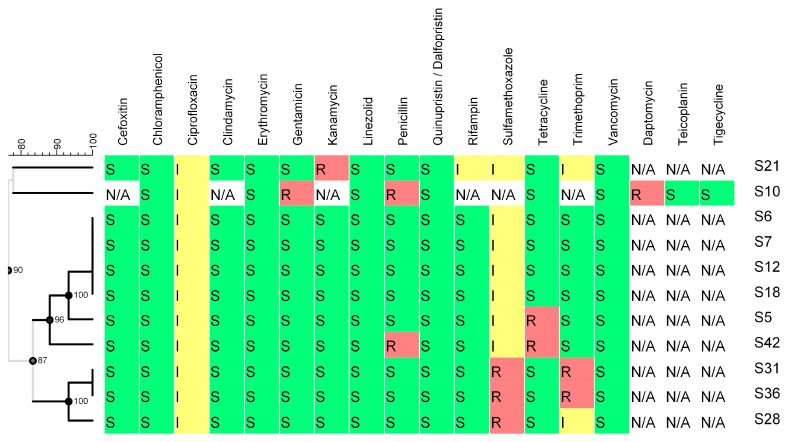
Antimicrobial resistance of selected strains of staphylococci. The type of resistance (S: sensitive, I: intermediate, and R: resistant) is colored according to the legend (green for S, yellow for I, and red for R). Hierarchical clustering was performed using the UPGMA method and the numbers indicate the calculated branch quality. N/A, this antibiotic was not tested for the corresponding strain. S5, S6, S7, S12, and S18: *S. aureus*; S21, S28, S31, and S36: *S. simulans*; S10: *S. sciuri*; S42: *S. epidermidis*.

The antimicrobial resistance of selected staphylococci was examined phenotypically (Figure 8). Some discrepancies in antimicrobial resistance (AMR) between the detection of ARGs and the actual phenotype were observed among strains, suggesting that AMR predictions relying on the in silico analysis of a sequenced genome should be always accompanied with AMR testing to reliably conclude the AMR of the microorganism [38]. The discrepancies were mainly related to the expression or not of the detected gene(s) during the AMR examination. For instance, the *tet38* gene, which encodes a tetracycline efflux pump, was detected in all *S. aureus* strains, but only the S5 strain showed resistance to the tetracycline antibiotic class. In addition, although genes conferring resistance to different antibiotic classes were detected in *S. aureus*, no strain presented an MDR phenotype. *Staphylococcus (Mammaliicoccus) sciuri* (S10) displayed an MDR pattern (resistant to multiple, three or more, different classes of antibiotics), which may have been attributed to the presence of the MDR phenotype, as predicted previously. Finally, *S. epidermidis* (S42) was also resistant to three different antibiotic categories (tetracycline-tetracyclines, penicillin-beta-lactams, and streptomycin-aminoglycosides), which aligned with the detected ARGs. The results for streptomycin are not shown in Figure 8 because there was no minimum inhibitory concentration (MIC) available for staphylococci; however, notably, *S. epidermidis* had higher streptomycin resistance (>32 mg/L) than the other tested strains (≤4 mg/L).

**Figure 9:**
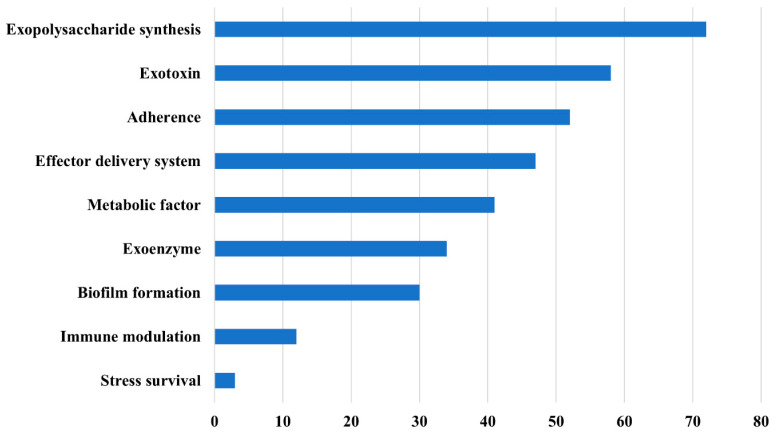
Distribution of virulence genes identified in staphylococci into functional categories.

We identified 62 VGs in the strains of *S. aureus* (*n* = 6) and two strains of *S. simulans*. Exopolysaccharide synthesis VGs were the most common (Figure 9). This category exclusively comprised type 8 capsular polysaccharide (*cap8*) operon genes (*cap8A*–*cap8G* and *cap8L*–*cap8P*). Type 8 is the most prevalent capsule type found in clinical *S. aureus* isolates, yet its exact role in virulence remains unclear and it most likely acts as a antiphagocytic factor [39]. 

**Figure 10:**
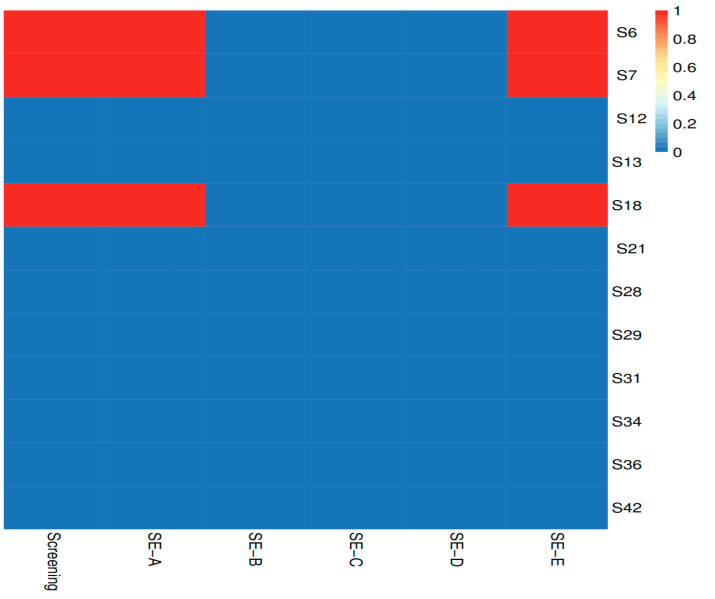
Staphylococcal enterotoxin production by selected species of staphylococci. Screening refers to application of the first test for enterotoxin A, B, C, D, or E production; SE-A, SE-B, SE-C, SE-D, and SE-E refer to application of the second test for specifying the production of the respective staphylococcal enterotoxin (SE), i.e., A, B, C, D, or E; red, enterotoxin-positive strains; blue, enterotoxin-negative strains; S6, S7, S12, and S18: *S. aureus*; S13, S21, S28, S29, S31, S34, and S36: *S. simulans*; S42: *S. epidermidis*.

Several genes encoding toxins were identified, shedding light on the potential virulence of staphylococci in dairy foods. The *hlb* and *hld* genes, which respectively encode beta- and delta-hemolysin, were found in six strains. This finding indicated that the staphylococcal strains analyzed in this study had the potential to cause hemolysis, which may have implications for host tissue damage and pathogenesis [40]. Moreover, we identified the *hlgA*, *hlgB*, and *hlgC* genes, which encode components of the gamma-hemolysin AB complex, as well as the alpha-hemolysin (*hla*) gene. Gamma- and alpha-hemolysins are associated with cytotoxicity and hemolytic activity and their presence in staphylococcal strains further highlighted their virulence potential and pathogenicity [41,42]. Furthermore, the *lukF-PV* gene (Panton–Valentine leukocidin F subunit) was detected. Panton–Valentine leukocidin (PVL) is a potent toxin that causes the lysis of leukocytes, while its presence has been strongly associated with the virulence of *S. aureus* strains [42]. Lastly, the *sea* gene, which encodes a heat-stable enterotoxin responsible for staphylococcal food poisoning, was found [43] in all *S. aureus* strains except the S12 strain. Interestingly, the *sak* gene, which advances microbial dissemination, was not found in the S12 strain either. The locus encoding staphylokinase (*sak*) usually contains additional virulence-related genes like enterotoxin A (*sea*) [44]. Phenotype testing confirmed that strain S12 was enterotoxin A-negative (Figure 10). The first screening test examined the production of enterotoxins A, B, C, D, or E and showed that only strains S6, S7, and S18 were able to produce staphylococcal enterotoxin (SE). The second specific test supported the previously obtained results specifying the SE production by the S6, S7, and S18 strains as enterotoxin A. The concurrent detection of enterotoxins A and E could be attributed to the occurrence of a cross-reaction that occurs between the antibody and toxin. However, the *see* gene (enterotoxin E) was not found in any of the *S. aureus* strains. Other enterotoxin-encoding genes detected in the *S. aureus* genomes were *seg*, *sei*, *sem*, *sen*, *seo*, and *seu*. Moreover, we were able to identify genes encoding adherence proteins. Among the identified adherence-related genes, *clfAB*, *ebp*, *fnbAB*, *sdrCD*, and *sdrEB* were of particular interest. These genes play a pivotal role in the regulation of staphylococcal adhesion to host tissues. The presence of these genes and the exoenzyme *aur* gene (encoding the metalloprotease aureolysin or protease III) suggested the ability of staphylococci to interact with host cells (immune evasion and dissemination) and likely contributes to their pathogenicity and persistence (survival) in various environmental niches [44,45].

Finally, it is worth mentioning the identification of genes related to biofilm formation. More specifically, we found multiple genes of the *ica* operon (*icaABCD* and *icaR*). This operon regulates the synthesis of polysaccharide intercellular adhesin (PIA), which is an essential component of biofilms formed by staphylococci, enabling adherence to surfaces as well as resistance to host immune responses [46]. 

The presence of virulence genes raises concerns regarding food safety and pathogenicity. Further investigation is needed to establish their expression levels and function along with their impact on the quality of dairy products.

#### 2.3.3. Bacteriocins (Auto-Inducing Peptides)

We found only one category of genes encoding bacteriocin-like molecules in staphylococci. Auto-inducing peptides (AIPs) I, II, and IV were identified in 13 strains of *S. aureus* and *S. simulans*. AIPs are a specific class of signaling molecules called quorum-sensing molecules. In staphylococci, AIPs play a crucial role in regulating various physiological processes, including VG and RG production and biofilm formation [47,48]. Moreover, AIPs seem to play a role in interspecies communication, thus enabling staphylococci to have interactions within microbial communities. These interactions can have implications for microbial pathogenesis, host–microbe interactions, as well as the overall balance of the microbiota [47].

#### 2.3.4. Mobile Genetic Elements and Prophages

Four plasmids were discovered in only two strains of our staphylococcal collection. The largest plasmid (29.2 Kbp), harbored by *S. aureus* strain S8, was a large, non-mobilizable plasmid with a GC content of 33.8%. The plasmid had high nucleotide identity with plasmid pSALNT106 (accession: CP042118), which was recently reported in *S. aureus* isolated from retail meat [49]. Notably, the authors identified phage proteins in pSALNT106, suggesting these plasmids may be implicated in the transmission of virulence factors [49]. The other three plasmids were all found in *S. lentus* strain S74. These were small, non-mobilizable plasmids (average size of 2.3 Kbp) that also had high nucleotide identity with plasmids in the aforementioned study, suggesting that a shared mobilome, largely encoding for phage proteins, exists between the staphylococcal strains of dairy and meat origin identified in the two studies.

MGEs were carried by *S. lentus*, *S. aureus*, *S. epidermidis*, and *Staphylococcus ureilyticus*, specifically MGEs *ISSep3*, *Tn559*, and *ISSau4*. *ISSep3* and *ISSau4* are ISs that are frequently found in *S. epidermidis* and *S. aureus*, respectively, and are highly associated with the transfer of RGs [50,51]. Lastly, we identified *Tn559*, a transposon that often carries the *dfrK* gene (trimethoprim resistance) in methicillin-resistant *S. aureus* [52]. As previously discussed, none of these genes (*dfrK* or *mec*-like) were present in our collection.

In the staphylococcal genomes, we confirmed the integration of 10 intact prophage genomes. *Staphylococcus* phage SA7 (accession: NC_048658.1) (found in *n* = 6 *S. simulans* genomes) was predominant, followed by *Staphylococcus* phage PT1028 (accession: AY954948.1) (*n* = 5). Prophages can harbor diverse genes that may promote staphylococcal virulence and antibiotic resistance [53]. Moreover, the integration of prophages can result in genomic rearrangements that further contribute to the genetic diversity of staphylococci. The presence of prophages in pathogens such as *S. aureus* can potentially enhance their pathogenicity or survival under different environmental conditions during the production of dairy products [54].

## 3. Materials and Methods

### 3.1. Microbial Strains and Culture Conditions

A collection of 39 microorganisms was used in this study, comprising two groups of LAB and staphylococci, which were all retrieved from raw sheep’s milk. The LAB species included *L. lactis* (*n* = 8), *L. petauri* (*n* = 6), *Lb. delbrueckii* subsp. *lactis* (*n* = 4), *Lmb. fermentum* (*n* = 2). The CNS comprised *S. simulans* (*n* = 8), *S. lentus*) (*n* = 1), *S. sciuri* (*n* = 1), *S. epidermidis* (*n* = 1), *S. saprophyticus* (*n* = 1), and *S. ureilyticus* (*n* = 1). Additionally, six strains of coagulase-positive *S. aureus* (*n* = 6) were also isolated. 

The LAB and staphylococci strains were stored at –80 °C in de Man, Rogosa and Sharpe (bacilli) (MRS, Madrid, Condalab, Spain, 1215) or M17 (cocci) broth (Himedia, Einhausen, Germany, M1029) and Brain Heart Infusion broth (BHI, Condalab, Madrid, Spain, 1400), respectively, with 30% glycerol (Penta Chemicals, Prague, Czech Rebublic, 14530–11000PE) as a cryoprotectant. Before use, the strains were resuscitated twice in the respective media for 24–48 h at 30 °C or 37 °C.

### 3.2. Whole Genome Sequencing, Assembly, and Quality Control

Bacterial DNA extraction and sequencing (Novaseq 6000 platform, Illumina, San Diego, CA, USA, 2 × 150 bp) was performed by Novogene Genomics Service (Novogene Co., Cambridge UK), as described in Syrokou et al. (2020) [55]. The quality of the adapter-free raw reads was checked using FastQC v.0.12.1 software available in the KBase platform [56,57]. Polishing and de novo assembling of the raw reads into contigs was performed using the Unicycler assembler and Pilon, respectively, provided by the BV-BRC v3.30.19a web platform [58,59,60]. Multi-Draft based Scaffolder (MeDuSa) v1.6 [61] was used to organize the contigs into scaffolds. Scaffolds were ordered and oriented based on the complete reference genomes present in the NCBI database (https://www.ncbi.nlm.nih.gov/, accessed on 10 January 2023); *Lactococcus lactis* LAC460, *Lactococcus petauri* B1726, *Lactobacillus delbrueckii* subsp. *jakobsenii* ZN7a-9 = DSM 26046, *Limosilactobacillus fermentum* SCB0035, *Staphylococcus aureus* NCTC 8325, and *Staphylococcus simulans* IVB6189. The CheckM tool v1.0.18 [62] of the KBase system was employed for quality evaluation of the scaffolds to ensure that the assembled genomes were of high quality, i.e., completeness (≥95%) and contamination (≤5%). Only one *S. aureus* genome had a completeness of 92.75% and contamination of 6.21%, but the genome was included in the downstream analysis because it was still of acceptable quality (i.e., completeness ≥ 90% and contamination ≤ 10%). Possible misassemblies after scaffolding were assessed by the mean of the Skew Index Test (SkweIT) v1.0 [63].

### 3.3. Genotyping and Comparative Genomics

The quality of genome assembly was evaluated using QUAST v5.2.0 [64]. Identification of species was carried out using Kraken2 v2.1.3 [65] and TYGS (https://tygs.dsmz.de/, accessed on 23 June 2023) [66]. ANI values between the strains’ genomes were estimated using OrthANI (https://www.ezbiocloud.net/, accessed on 23 June 2023) [67] and a heatmap of the resulted ANI values was constructed using MORPHEUS (https://software.broadinstitute.org/morpheus/, accessed on 09 August 2023). The annotation of genomes was performed using PROKKA v1.14.5 [68], whereas the functional annotation of open reading frames and analysis of subsystems were performed using eggnog-mapper v2.1.12 [69]. Additionally, prophages were identified using the PHASTEST API service (https://phastest.ca/, accessed on 27 June 2023) [70]. Abricate v1.0.0 [71] along with the reference databases VFDB v1.0 [72], MobileElementFinder v1.1.2 [73], Resfinder v2.1 [74], and PlasmidFinder v2.2 [75] were used to establish the occurrence of VGs, MGEs, RGs, and plasmid replicons, respectively. Furthermore, bacteriocins were detected using the BAGEL4 webserver (http://bagel5.molgenrug.nl/, accessed on 27 June 2023), whereas CAZymes were identified using the dbCAN2 server (https://bcb.unl.edu/dbCAN2/, accessed on 27 June 2023) [76]. ST classification and *spa* typing for *S. aureus* strains were carried out using Center for Genomic Epidemiology (CGE) services (http://www.genomicepidemiology.org/services/, accessed on 27 June 2023), i.e., MLST 2.0 [77] and spaTyper 1.0 [78], respectively. Finally, resistance to methicillin (detection of Staphylococcal Cassette Chromosome Elements—SCC *mec*) and pathogenicity of staphylococci were evaluated using CGE services SCCmecFinder 1.2 [79] and PathogenFinder 1.1 [80], respectively.

Roary v3.11.2 [81] software was utilized to conduct pangenome analysis and core genome alignment of both LAB and staphylococci. Proteins were classified into the same family if their amino acid sequence similarity was equal to or greater than 75%. For a gene to be considered a core gene, it needed to be present in at least 90% of the isolates. To visualize the phylogenetic relationship, the FastTree v2.1 algorithm [82] was employed, and the resulting tree was annotated and visualized using iTOL v6 [83]. The European public Galaxy server (https://usegalaxy.eu/, accessed on 27 June 2023) [84] was used for some of the aforementioned analyses. Finally, predictions regarding LAB probiotic status were made using the iProbiotics webtool (http://bioinfor.imu.edu.cn/iprobiotics/public/Home, accessed on 9 August 2023).

### 3.4. Phenotype Testing

#### 3.4.1. Antimicrobial Resistance

Selected strains of staphylococci were tested for AMR using the Sensititre MIC (Thermo Fisher Scientific, Waltham, MA, USA) technique employing the broth microdilution MIC approach, according to manufacturer’s instructions. The AMR plates used were the Sensititre EU Surveillance Staphylococcus EUST AST Plate and Sensititre EU Surveillance Enterococcus EUVENC AST Plate (Thermo Fisher Scientific). The results were presented in a dendrogram using Bionumerics v8.1 software and the antibiotic susceptibility plugin (bioMérieux, Sint-Martens-Latem, Belgium). The strains were classified as susceptible (S), resistant (R) or intermediate (I) based on the European Committee on Antimicrobial Susceptibility Testing (EUCAST) Breakpoint Table v13.0 for *Staphylococcus* spp. (https://www.eucast.org/clinical_breakpoints/, accessed on 10 January 2023).

#### 3.4.2. Staphylococcal Enterotoxin Production

Selected staphylococci strains were studied for the production of enterotoxin(s) (A, B, C, D, or E) using the Ridascreen Set Total assay kit (R-Biopharm, Darmstadt, Germany, R4105) (screening test) and the Ridascreen Set A, B, C, D, E assay kit (R-Biopharm, Darmstadt, Germany, R4101) (specific test), according to manufacturer’s instructions. A heatmap was constructed with the obtained results using ImageGP software (https://www.bic.ac.cn/ImageGP/, accessed on 9 August 2023) [85].

#### 3.4.3. Probiotic Properties

LAB (*L. petauri*, *L. lactis*, *Lb. delbrueckii* subsp. *bulgaricus*, and *Lmb. fermentum*) microbial cells were harvested at 3350 *× g* for 15 min at 4 °C (Centrifuge 5804 R, Eppendorf, Oslo, Norway), washed twice with sterile Ringer solution (Sigma-Aldrich, Taufkirchen, Germany, 96724), and resuspended using the same dilutor until an optical density (OD) of 0.6–0.7 was achieved at 600 nm (BioTek Instruments – Agilent Technologies, Epoch, Santa Clara, CA, USA) (*A_0_*). Afterwards, two milliliters of the bacterial suspension were mixed with two milliners of xylene (Fisher Scientific, Loughborough, Leicestershire, UK, X/0250/17) and homogenized with vortexing. Aliquots of 150 μL of each bacterial suspension were transferred to a 96-well microplate (Thermo Fisher Scientific, Lillerød, Denmark, 167008) and left for 0.5 h at room temperature (*A*). The cell surface hydrophobicity was calculated using the following equation [86]:

(1) $$cell\;surface\;hydrophobicity\;\left(\%\right)=\left[\frac{\left(A_{0}-A\right)}{A_{0}}\right]\times 100$$

where *A_0_* and *A* are the initial (0 h) and final (after 0.5 h) OD_600_, respectively.

For the auto-aggregation assay, samples in the 96-well microplate were incubated at 37 °C for 5 h and the OD_600_ was measured every 1 h (*A_t_*). The auto-aggregation was estimated as follows [76]:

(2) $$auto-aggregation\;\left(\%\right)=\left[1-\frac{A_{t}}{A_{0}}\right]\times 100$$

where *A_t_* is the measured OD_600_ at each time interval (1, 2, 3, 4, and 5 h).

Both assays were carried out in quadruplicate. Statistical comparisons were performed using GraphPad Prism v9.5.1 software (GraphPad Software, Boston, MA, USA) using the unpaired multiple parametric *t*-test with Welch’s correction, i.e., no assumption was made about consistent SDs between species (columns) for each cell surface assay (row), and the false discovery rate (FDR) approach (two-stage linear step-up method of Benjamini, Krieger, and Yekutieli), i.e., adjusted *p*-value (*q*-value = 0.05) for multiple comparisons.

## 4. Conclusions

Τhe in silico characterization of staphylococcal and LAB strains isolated from raw sheep’s milk revealed the presence of various genetic elements, notably MGEs and specific genes associated with AMR, virulence, and metabolic capabilities. These findings hold practical implications for dairy product safety and handling.

The presence of MGEs, including plasmids, transposons, and IS elements, underscores the importance of safe dairy production practices. Since raw sheep’s milk seems to serve as a reservoir for these genetic elements, the necessity for tight measures to ensure the safety of dairy products should be emphasized. Therefore, implementation of rigorous pasteurization and quality control procedures is crucial to mitigate the risk of transmitting AMR and potential foodborne pathogens to consumers. Moreover, concerns arise regarding the potential transfer of RGs and VGs from staphylococci to LAB. While LAB are generally recognized as safe (GRAS), it is essential to continuously monitor the genetic dynamics between these bacterial groups. Further research is warranted to investigate gene transfer events in order to ensure that LAB maintain their safe status and can continue to be used for their probiotic properties. This knowledge can guide the development of safer and more effective probiotics for dairy product applications.

## Figures and Tables

**Table 1:** Identification of species and assembly statistics for all genomes of this study. All strains were isolated from raw sheep’s milk.

Strain ID	Species	Genome Size (Mbp)	GC Content (%)	No. of Scaffolds	N50 (Mbp)	No. of CDSs
S87	*Limosilactobacillus fermentum*	1.86	52.71	3	1.52	1765
S94	*Limosilactobacillus fermentum*	1.83	52.63	2	1.57	1746
S54	*Lactobacillus delbrueckii* subsp. *lactis*	1.92	49.69	3	0.60	1794
S70	*Lactobacillus delbrueckii* subsp. *lactis*	1.91	49.71	2	1.91	1791
S88	*Lactobacillus delbrueckii* subsp. *lactis*	1.85	49.94	2	1.85	1725
S96	*Lactobacillus delbrueckii* subsp. *lactis*	1.91	49.7	4	0.66	1790
S43	*Lactococcus lactis*	2.27	35.06	1	2.27	2052
S44	*Lactococcus lactis*	2.27	35.06	1	2.27	2051
S49	*Lactococcus lactis*	2.25	35.12	1	2.25	2053
S58	*Lactococcus lactis*	2.25	35.12	1	2.25	2053
S71	*Lactococcus lactis*	2.35	35	2	2.27	2103
S73	*Lactococcus lactis*	2.45	34.99	3	1.28	2232
S76	*Lactococcus lactis*	2.32	34.93	2	2.20	2098
S9	*Lactococcus lactis*	2.27	35.06	1	2.27	2052
S27	*Lactococcus petauri*	1.96	38.19	2	1.77	1864
S46	*Lactococcus petauri*	1.95	38.19	2	1.76	1848
S77	*Lactococcus petauri*	1.95	38.23	2	1.76	1839
S78	*Lactococcus petauri*	1.96	38.2	2	1.77	1856
S82	*Lactococcus petauri*	1.92	38.26	2	1.70	1820
S84	*Lactococcus petauri*	1.96	38.2	2	1.77	1855
S12	*Staphylococcus aureus*	2.75	32.78	2	1.50	2565
S18	*Staphylococcus aureus*	2.75	32.78	2	1.39	2379
S5	*Staphylococcus aureus*	2.80	32.74	3	2.76	2536
S6	*Staphylococcus aureus*	2.75	32.78	1	2.75	2534
S7	*Staphylococcus aureus*	2.75	32.79	3	2.64	2579
S8	*Staphylococcus aureus*	2.87	32.93	3	2.42	2531
S42	*Staphylococcus epidermidis*	2.32	32.05	1	2.32	2534
S74	*Staphylococcus lentus*	2.58	31.79	48	0.17	2594
S85	*Staphylococcus saprophyticus*	2.56	33.02	1	2.56	2153
S10	*Staphylococcus sciuri*	2.40	32.41	2	2.17	2510
S13	*Staphylococcus simulans*	2.68	35.89	7	2.65	2531
S21	*Staphylococcus simulans*	2.66	35.87	11	0.66	2582
S28	*Staphylococcus simulans*	2.36	35.96	1	2.36	2278
S29	*Staphylococcus simulans*	2.57	36.03	10	0.55	2425
S31	*Staphylococcus simulans*	2.23	36.12	1	2.23	2141
S34	*Staphylococcus simulans*	2.57	36.05	2	1.33	2396
S36	*Staphylococcus simulans*	2.57	36.05	1	2.57	2396
S98	*Staphylococcus simulans*	2.57	36.04	1	2.57	2388
S99	*Staphylococcus ureilyticus*	2.53	32.51	2	2.49	2379

Abbreviations: Mbp; megabase pair, GC content; guanine-cytosine content, N50; 50% of the entire assembly is contained in contigs equal to or larger than this value, CDSs; coding sequences.

**Table 2:** Probiotic marker genes identified in the 20 LAB strains of this study.

Function	Gene	Number of LAB Strains
Acid stress	*atpABCDEFGH*	20/20
	*gadB*	14/20
	*nhaC*	12/20
Adhesion	*eno, lspA, srtA, tpiA, tuf*	20/20
	*epsB*	6/20
	*mapA*	16/20
	*pgi*	18/20
Antioxidant	*fnr*	3/20
	*mntH, nrdH*	16/20
	*msrAB, trxAB*	20/20
	*ndh*	14/20
	*nox*	1/20
	*npr*	2/20
	*poxL*	8/20
	*tpx*	10/20
Bile tolerance	*cfa*	16/20
	*ppaC*	20/20
Cold stress	*cspA*	18/20
Heat stress	*clpB*	14/20
	*clpCEPX, dnaJK, grpE, hrcA, hslO, lon*	20/20
	*clpL, hslUV*	4/20
	*ctsR*	16/20
	*htpX*	6/20
Immunomodulation	*dltABCD*	20/20

## Data Availability

The whole-genome sequencing data have been deposited at GenBank (NCBI) under accession (BioProject) number PRJNA1007360 (https://www.ncbi.nlm.nih.gov/).

## Supplementary Materials

The following supporting information can be downloaded at: https://www.mdpi.com/article/10.3390/ijms241813883/s1.
